# Analysis of Class I Major Histocompatibility Complex Gene Transcription in Human Tumors Caused by Human Papillomavirus Infection

**DOI:** 10.3390/v9090252

**Published:** 2017-09-10

**Authors:** Steven F. Gameiro, Ali Zhang, Farhad Ghasemi, John W. Barrett, Anthony C. Nichols, Joe S. Mymryk

**Affiliations:** 1 Department of Microbiology and Immunology, The University of Western Ontario, London, ON N6A 3K7, Canada; sgameiro@uwo.ca (S.F.G); a.zhanger@gmail.com (A.Z.); 2 Department of Otolaryngology, Head & Neck Surgery, The University of Western Ontario, London, ON N6A 3K7, Canada; fghasemi2019@meds.uwo.ca (F.G.); john.barrett@lhsc.on.ca (J.W.B.); Anthony.Nichols@lhsc.on.ca (A.C.N.); 3 Department of Oncology, The University of Western Ontario, London, ON N6A 3K7, Canada; 4 London Regional Cancer Program, Lawson Health Research Institute, London, ON N6C 2R5, Canada

**Keywords:** human papillomavirus, MHC-I, major histocompatibility complex, antigen presentation, immune evasion, head & neck carcinoma, cervical carcinoma

## Abstract

Oncoproteins from high-risk human papillomaviruses (HPV) downregulate the transcription of the class I major histocompatibility complex (MHC-I) antigen presentation apparatus in tissue culture model systems. This could allow infected or transformed cells to evade the adaptive immune response. Using data from over 800 human cervical and head & neck tumors from The Cancer Genome Atlas (TCGA), we determined the impact of HPV status on the mRNA expression of all six MHC-I heavy chain genes, and the β2 microglobulin light chain. Unexpectedly, these genes were all expressed at high levels in HPV positive (HPV+) cancers compared with normal control tissues. Indeed, many of these genes were expressed at significantly enhanced levels in HPV+ tumors. Similarly, the transcript levels of several other components of the MHC-I peptide-loading complex were also high in HPV+ cancers. The coordinated expression of high mRNA levels of the MHC-I antigen presentation apparatus could be a consequence of the higher intratumoral levels of interferon γ in HPV+ carcinomas, which correlate with signatures of increased infiltration by T- and NK-cells. These data, which were obtained from both cervical and oral tumors in large human cohorts, indicates that HPV oncoproteins do not efficiently suppress the transcription of the antigen presentation apparatus in human tumors.

## 1. Introduction

Anti-viral immunity is comprised of multiple levels of defenses that cooperatively block, control, and eliminate infection. Intrinsic and innate immunity function as pre-existing defenses against viral infection, and serve as the first levels of response [1,2]. In the event that these non-specific immune responses are not sufficient to eliminate an infection, subsequent antigen-specific adaptive immune responses develop. As such, the recognition of intracellularly-derived viral peptides in the context of major histocompatibility complex class I (MHC-I) molecules by cytotoxic T lymphocytes (CTLs) triggers the elimination of virally infected cells [3], while antibodies specific for virion antigens neutralize viral particles, and block the spread of infection [4].

Despite the effectiveness of the immune system, many viruses have acquired sophisticated methods to evade anti-viral defenses, including the adaptive CTL response. For example, several viral proteins have been identified that block MHC-I antigen presentation by downregulating the loading of antigen onto MHC-I, the expression of MHC-I itself, and the transport of MHC-I to the cell surface. Viral proteins have also been found to trigger MHC-I internalization and/or degradation [5]. Collectively, these strategies facilitate the evasion of the infected cell from CTL attack, thus allowing the virus to persist.

Human papillomaviruses (HPV) are small, non-enveloped, double-stranded DNA viruses that infect mucosal or cutaneous epithelia and induce cellular proliferation [6]. HPV infections of mucosal tissues are highly prevalent, and infections typically take one to two years to resolve [7]. Mucosal HPVs are classified as low- or high-risk based on the frequency with which they are associated with cancer. High-risk HPVs are causative agents for cancers at multiple distinct anatomical subsites [6], including the cervix [8], other anogenital tissues [9,10,11], and the oropharynx [12,13,14]. Worldwide, mucosal high-risk HPV infection is responsible for greater than 5% of all human cancers [15].

HPV encodes two main oncogenes, E6 and E7 [16], which are constitutively expressed in HPV positive (HPV+) tumors. The E6 and E7 oncoproteins perform multiple functions in an infected and/or cancerous cell. They interact with key cellular regulatory proteins to dysregulate gene expression and cell growth [16]. For example, E6 binds to and degrades p53, while E7 targets the retinoblastoma protein (Rb) and its family members [17,18]. These events contribute to inappropriate cell cycle progression in HPV-infected cells [19,20]. In high-risk HPV infections, viral oncoproteins uncouple cell growth and differentiation. Consequently, this contributes to the formation of epithelial dysplasia, which may progress to carcinoma if the infection is not resolved [16].

Multiple studies have suggested that HPV oncoproteins contribute to the evasion of the adaptive immune response, which supports a model by which the establishment of a persistent HPV infection contributes to the ability of HPV+ cancers to evade anti-tumor CTL responses [21]. Although both E5, a third viral oncoprotein and E6 from HPV16 have been reported to block MHC-I expression [22,23], the majority of studies in this area focus on the E7-mediated reduction of MHC-I expression. Specifically, transfection of high-risk HPV E7 into various cell lines represses MHC-I and/or transporter associated with antigen processing (TAP) expression [24,25,26]. Reciprocal studies have shown that the knock-down of E7 in some HPV+ cervical cancer cell lines increases MHC-I expression [27,28]. In these studies, the repression of MHC-I appears to be a specific function of high-risk HPV E7 that is not shared by low-risk HPV E7 [24,25]. Mechanistically, the reduction of MHC-I and TAP expression in these studies occurs at the transcriptional level [24,25,26]. The most detailed studies of the HPV-mediated repression of MHC-I expression report that E7 associates with the promoter of the MHC-I HLA-A heavy chain gene, and recruits histone deacetylase activity via residues 70, 80, and 88 of HPV16 E7 [25,28,29]. In addition to repressing the basal transcription of MHC-I, HPV E7 abrogates the interferon regulatory factor-1 (IRF-1) response, thus inhibiting interferon γ (IFN-γ)-mediated transcriptional induction of MHC-I expression, antigen presentation, and CTL-induced cell death [30,31,32,33,34].

While the majority of published studies using tissue culture models indicate that HPV oncoproteins such as E7 repress the expression of the antigen presentation apparatus, cognate studies on the effects of HPV in human carcinomas are less consistent. Conflicting reports suggest that HPV+ carcinomas/dysplastic lesions display increased [35,36], decreased [37,38,39,40], or the same levels [41,42] of MHC-I or TAP expression when compared with HPV negative (HPV−) tumors or normal control tissues. Some of these differences may be related to different tissue types, stages, and/or grades. The most comprehensive analysis, which examined 109 cervical carcinoma samples, concluded that only about 40% of the samples exhibited MHC-I downregulation [43]. However, many types of tumors that are HPV− contain a similar subset that are deficient in MHC-I and/or TAP expression [44], which suggests that this reduction is not actually virally-mediated. As such, it remains an open question as to whether the E7-mediated changes in the transcription of various MHC-I pathway components observed in tissue culture similarly occur in the context of actual human HPV+ carcinomas. This information is clearly relevant, considering that the activation of an antigen-dependent clearance of tumor cells by the immune system is an extraordinarily effective cancer therapy [45].

In this study, we used data from over 800 human cervical [46] and head & neck tumors [47] from The Cancer Genome Atlas (TCGA). We set out to determine if high-risk HPV oncogene expression alters the expression of MHC-I heavy chain genes, the β2 microglobulin light chain gene, and other components of the antigen-loading apparatus, which includes: TAP1/2, tapasin, ERp57, calreticulin, calnexin, and ERAP1/2 in human tumors. We found that HPV+ tumors from both anatomical sites expressed substantial levels of mRNA for each of these MHC-I pathway components. Indeed, many of these genes were expressed at significantly higher levels than normal control tissues. Furthermore, an analysis of mutation frequencies in MHC-I genes indicated that the HPV+ and HPV− cancers exhibit similar frequencies of alteration. This is in contrast to TP53 and CDKN2A, which are key regulators of pathways known to be targeted by HPV oncoproteins, and are frequently mutated in HPV− but not HPV+ carcinomas. This suggests that the acquisition of somatic mutations in MHC-I genes as a means of evading the anti-tumor CTL response occurs similarly in HPV+ and HPV− cancers, which would not be expected if HPV oncoproteins were effective at repressing MHC-I expression or function. Taken together, this data does not entirely validate conclusions drawn from previous tissue culture studies. Specifically, HPV oncoprotein expression does not repress the transcription of the MHC-I pathway components in cervical carcinomas, although a modest reduction in MHC-I mRNA levels was observed in HPV+ vs. HPV− head & neck cancers.

## 2. Materials and Methods

### 2.1. RNA Expression Comparisons and Statistical Analysis

Level 3 RNA-Seq by Expectation Maximization (RSEM) normalized Illumina HiSeq RNA expression data for the TCGA head & neck cancer (HNSC) and cervical carcinoma (CESC) cohorts was downloaded from the Broad Genome Data Analysis Center’s Firehose server (https://gdac.broadinstitute.org/). For all of the genes, the gene level Firehose dataset was used. Normalized expression data was extracted into Microsoft Excel, and the HPV status was manually curated based on published datasets [46,47,48,49]. For each gene analyzed, primary patient samples with known HPV status were grouped as HPV+, HPV−, or normal control tissue. Patient samples with unknown HPV status were omitted from our calculations, as were samples obtained from secondary metastatic lesions. This resulted in 73 HPV+, 442 HPV−, and 43 normal control samples with data available for the HNSC gene expression analysis and 278 HPV+, 19 HPV−, and 3 normal control samples available for the CESC gene expression analysis. Boxplot comparison of gene expression was performed using GraphPad Prism v7.0 (Graphpad Software, Inc., San Diego, California, USA) and assembled into final form using CorelDRAW (Corel, Ottawa, Ontario, Canada). For the boxplots, center lines show the medians, box limits indicate the 25th and 75th percentiles as determined by Graphpad Prism, and whiskers extend from the minimum to maximum values. Statistical significance was calculated using Graphpad Prism. *p*-values were assigned using a one-tailed non-parametric Mann–Whitney U test. Post-hoc power calculations were performed with G*Power software version 3.1.9.2 [50], using post-hoc *t*-test family calculations, with effect size selected as 0.8 and α = 0.05. All comparisons achieved a power value >0.8, or demonstrated significant differences, unless otherwise noted in the text.

### 2.2. Somatic Mutation Frequency Comparisons and Statistical Analysis

Somatic mutations present in selected genes of the tumor samples in the TCGA, HNSC, and CESC cohorts were downloaded as the DNA Mutation Packager Calls dataset from the Broad Genome Data Analysis Center’s Firehose server (https://gdac.broadinstitute.org/), and manually annotated for HPV status. This resulted in 67 HPV+ and 431 HPV− samples for the HNSC cohort, and 181 HPV+ and 10 HPV– samples for the CESC cohort. Differences in the frequencies of DNA mutations in selected genes were calculated between HPV+ and HPV− samples using the maftools package [51] in R statistical environment (Version 3.4.0). Silent mutations were excluded from the analysis, and samples with multiple mutations in a gene were counted as one for the calculations. Statistical significance (*p* < 0.05) was determined using Fisher’s exact test.

## 3. Results

### 3.1. Impact of HPV Status on MHC-I Heavy Chain Expression in Human Tumors

**Figure 1:**
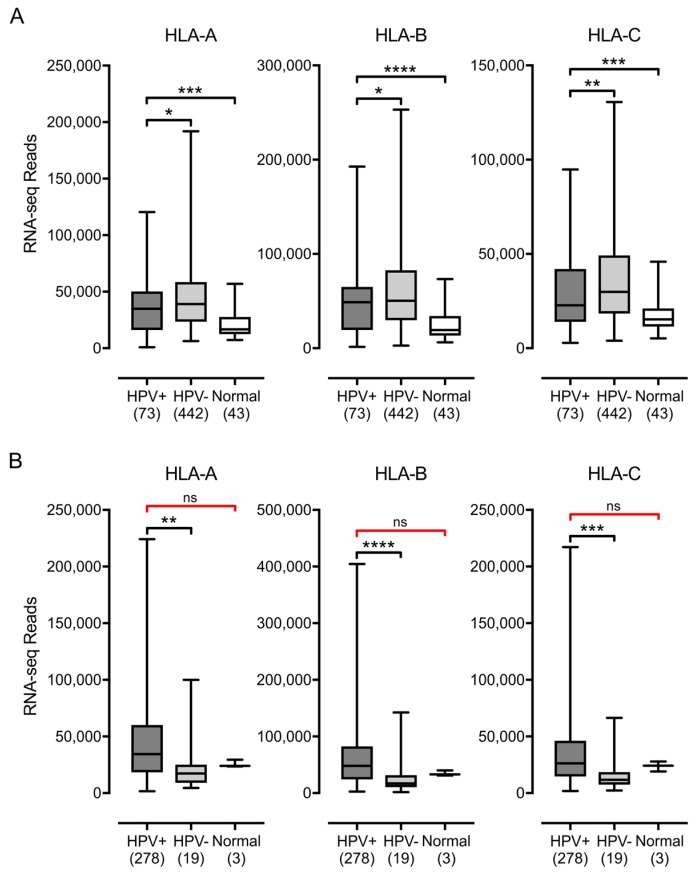
Expression of classical major histocompatibility complex class I (MHC-I) heavy chain genes in head & neck or cervical carcinomas stratified by human papillomavirus (HPV) status. Normalized RNA-seq data for the indicated MHC-I heavy chain genes was extracted from The Cancer Genome Atlas (TCGA) database for the head & neck cancer (HNSC) (**A**) and cervical carcinoma (CESC) (**B**) cohorts for HPV+, HPV−, and normal control tissues. Numbers in brackets refer to the number of samples included in each analysis. * *p* ≤ 0.05; ** *p* ≤ 0.01; *** *p* ≤ 0.001; **** *p* ≤ 0.0001; ns—not significant; red brackets indicate a comparison that did not achieve significance with a power value <0.8.

MHC-I is a heterodimer comprised of a heavy chain encoded by one of three classical (HLA-A, -B, and -C) or non-classical (HLA-E, -F, and -G) genes and the invariant β2 microglobulin light chain [5]. We analyzed the Illumina HiSeq RNA expression data from the TCGA head & neck squamous cell carcinoma (HNSC) and cervical carcinoma (CESC) cohorts for expression of HLA-A, HLA-B, and HLA-C, the three classical genes (Figure 1). Unexpectedly, HPV+ samples from both the HNSC and CESC cohorts expressed high levels of mRNA for all three genes, as shown by the normalized RNA-seq absolute read count values, which averaged in the range of 25,000–50,000. The HPV+ samples from the HNSC cohort expressed significantly elevated levels of HLA-A, HLA-B, and HLA-C compared with normal control tissue. In the CESC cohort, although the median RNA-seq values for the HPV+ samples appeared at least as high as those for normal control samples, there was insufficient power to exclude the possibility that the expression of HLA-A, HLA-B, or HLA-C was not different. In the HNSC cohort, levels in HPV+ samples were slightly lower than HPV− samples, whereas HPV+ samples in the CESC cohort had substantially higher levels of expression versus HPV− samples. Nevertheless, all tumor subsets expressed high absolute levels of the mRNAs for each of the classical MHC-I heavy chain genes.

**Figure 2:**
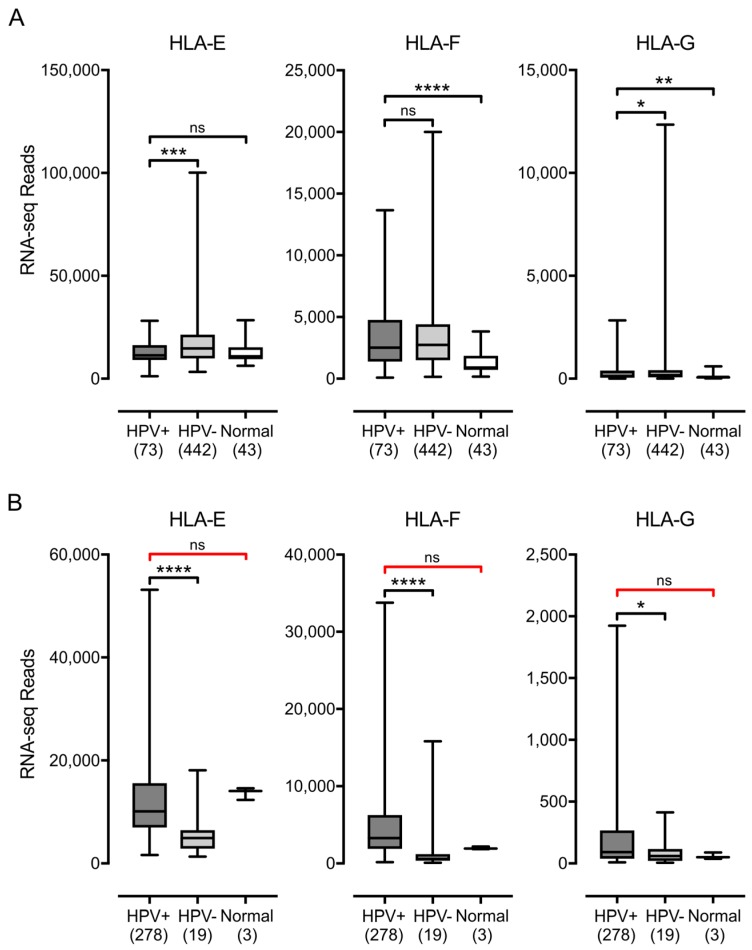
Expression of non-classical MHC-I heavy chain genes in head & neck or cervical carcinomas stratified by HPV status. Normalized RNA-seq data for the indicated MHC-I heavy chain genes was extracted from the TCGA database for the HNSC (**A**) and CESC (**B**) cohorts for HPV+, HPV−, and normal control tissues. Numbers in brackets refer to the number of samples included in each analysis. * *p* ≤ 0.05; ** *p* ≤ 0.01; *** *p* ≤ 0.001; **** *p* ≤ 0.0001; ns—not significant; red brackets indicate a comparison that did not achieve significance with a power value <0.8.

Similarly, higher or comparable levels of expression of the non-classical genes, HLA-E, HLA-F, and HLA-G, were observed in HPV+ cancers with respect to normal control tissues in the HNSC cohort (Figure 2). Again, a comparison of the HPV+ samples to normal control samples in the CESC cohort was insufficiently powered to allow us to exclude the possibility that the expression of HLA-E, HLA-F or HLA-G was not altered by the presence of HPV oncogenes. However, HLA-E and HLA-F were expressed at relatively high levels based on the absolute normalized read values. As noted by others [52], HLA-E was expressed at significantly reduced levels in HPV+ samples compared with HPV− samples in the HNSC cohort. However, this was not a consistent effect in all HPV+ carcinomas, as HLA-E was expressed at much higher levels in the HPV+ samples compared with the HPV− samples in the CESC cohort. Although HLA-G was expressed at lower overall levels than the other heavy chain genes, it was also expressed at comparable or elevated levels in HPV+ samples versus HPV− or normal control samples (Figure 2). Collectively, these results indicate that the expression of HPV oncogenes in actual human tumors is not correlated with strong repression of the MHC-I loci, in contrast to what is reported in tissue culture based models. Indeed, the average expression of the mRNA for these genes is consistently higher or equivalent in HPV+ samples versus the normal control.

### 3.2. Impact of HPV Status on the Expression of other Components of the Antigen Presentation Apparatus in Human Tumors

**Figure 3:**
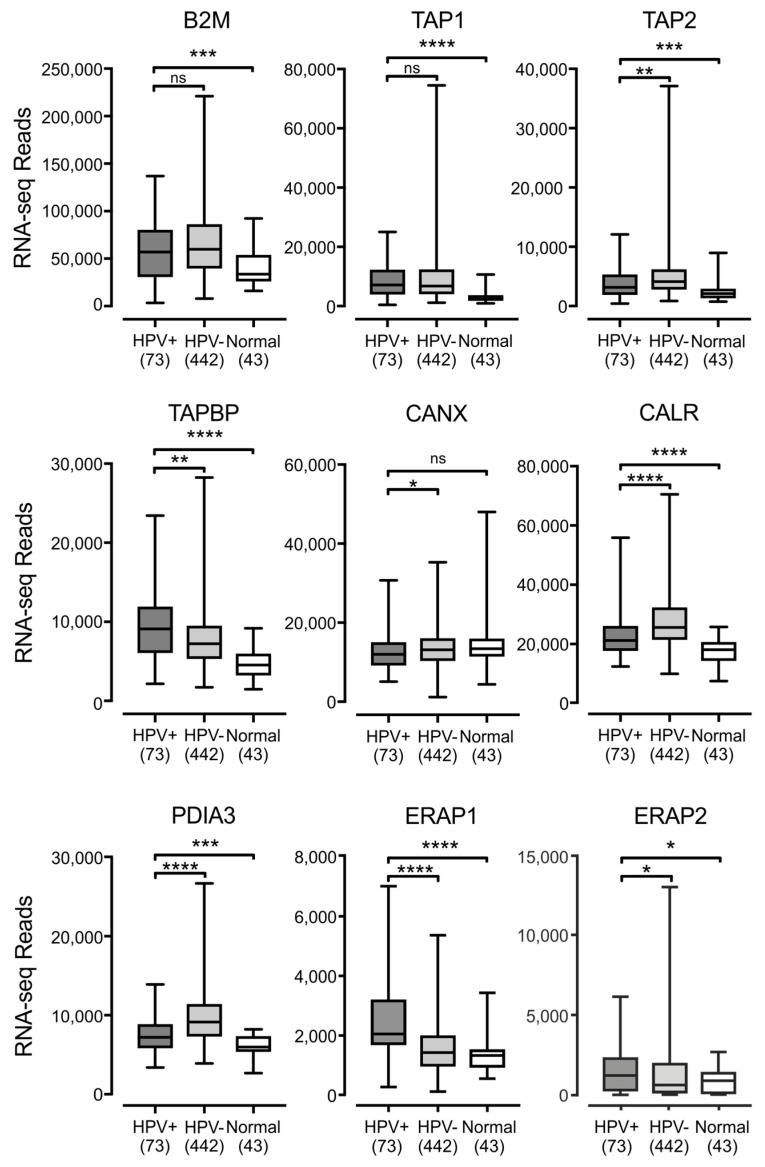
Expression of the MHC-I light chain and other genes involved in MHC-I dependent antigen presentation in head & neck carcinomas stratified by HPV status. Normalized RNA-seq data for the indicated genes involved in MHC-I dependent antigen presentation was extracted from the TCGA database for the HNSC cohort for HPV+, HPV−, and normal control tissues. Numbers in brackets refer to the number of samples included in each analysis. * *p* ≤ 0.05; ** *p* ≤ 0.01; *** *p* ≤ 0.001; **** *p* ≤ 0.0001; ns—not significant.

**Figure 4:**
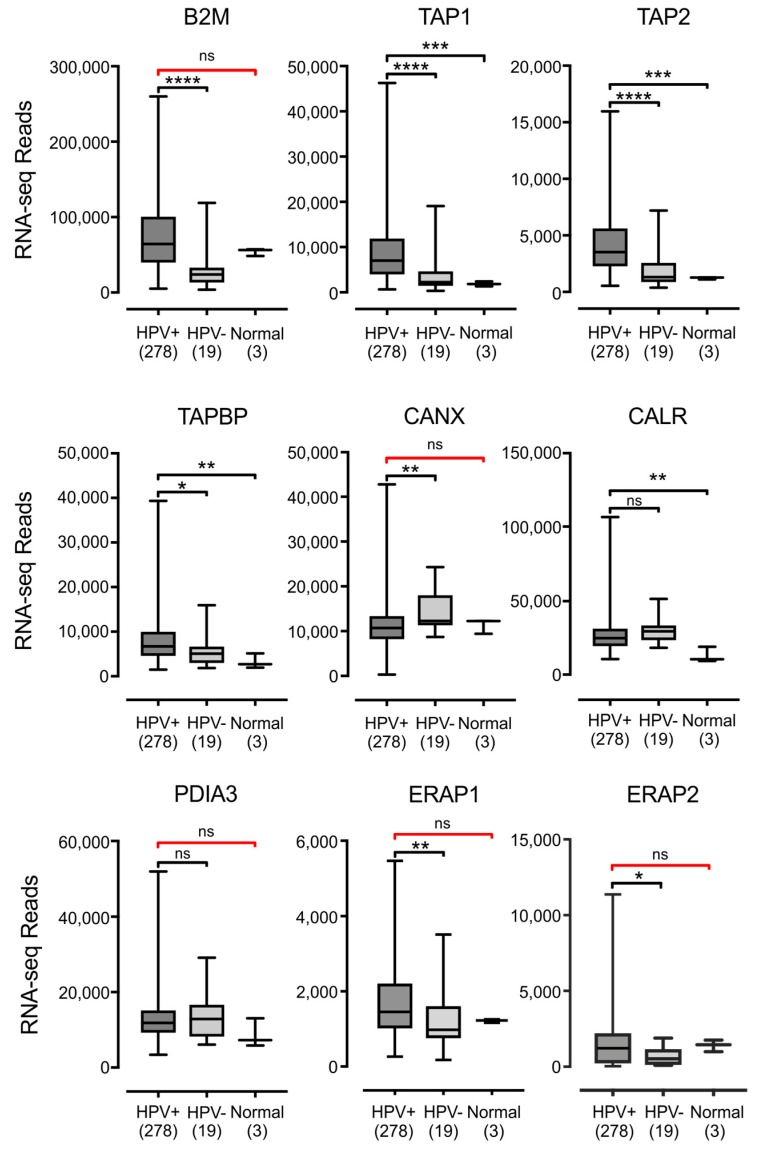
Expression of the MHC-I light chain and other genes involved in MHC-I dependent antigen presentation in cervical carcinomas stratified by HPV status. Normalized RNA-seq data for the indicated genes involved in MHC-I dependent antigen presentation was extracted from the TCGA database for the CESC cohort for HPV+, HPV−, and normal control tissues. Numbers in brackets refer to the number of samples included in each analysis. * *p* ≤ 0.05; ** *p* ≤ 0.01; *** *p* ≤ 0.001; **** *p* ≤ 0.0001; ns—not significant; red brackets indicate a comparison that did not achieve significance with a power value <0.8.

The MHC-I heavy chain folds and assembles with the β2 microglobulin light chain within the endoplasmic reticulum, in a process dependent on binding to a peptide antigen [5]. The MHC-I peptide-loading complex consists of MHC-I, the peptide transporter TAP, the bridging factor tapasin, the endoplasmic reticulum aminopeptidase (ERAP), and the chaperones: calreticulin, calnexin, and ERp57. Several studies have reported the downregulation of TAP in HPV+ cancer cell lines [24,26]. Much less is known about the effect of HPV status on the expression of the other components necessary for MHC-I antigen presentation, so we examined the impact of HPV status on all mRNA expression for all components of the MHC-I peptide-loading complex. Analysis of TCGA data revealed high levels of transcripts for the B2M gene encoding β2 microglobulin, the TAP1 and TAP2 genes encoding the TAP heterodimer, and the genes encoding ERAP, tapasin, calreticulin, calnexin, and ERp57 (ERAP1/2, TAPBP, CALR, CANX, and PDIA3 respectively) in HPV+ samples in head & neck (Figure 3) and cervical (Figure 4) carcinomas. All genes were expressed at higher levels in HPV+ samples with respect to normal control tissue in the HNSC cohort, with the sole exception of CANX, which was expressed at a comparable level. There were no consistent differences in expression with respect to HPV+ and HPV− samples in the HNSC cohort. B2M and TAP1 were expressed at equivalent levels; TAP2, CANX, CALR, and PDIA3 were reduced; while TAPBP, ERAP1, and ERAP2 were increased (Figure 3). In the CESC cohort, nearly all of these genes were expressed at elevated or comparable levels to normal control tissue or HPV− samples, although some comparisons were limited based on insufficient sample numbers. The sole exception was CANX, which was expressed at a lower level in HPV+ versus HPV− samples (Figure 4). Thus, most components of the MHC-I loading complex were transcribed in HPV+ human tumors at levels that appear similar or significantly higher than normal control tissue. This again contrasts with the reported decreases in TAP transcription reported in tissue culture models.

### 3.3. Higher Levels of Lymphocytes and Interferon γ Are Present in HPV+ Human Tumors

IFN-γ, the product of the IFNG locus, can coordinately induce higher levels of transcription in many of the genes involved in antigen processing and presentation by MHC-I, and provides enhanced immune surveillance under inflammatory conditions [53]. Given our observation that mRNA levels of all MHC-I loading and presentation genes were expressed at higher or at least similar levels in HPV+ tumors compared with HPV− and normal control tissues, we hypothesized that infiltrating lymphocytes producing IFN-γ could be present at higher levels in HPV+ tumors versus HPV− tumors or normal control tissues.

**Figure 5:**
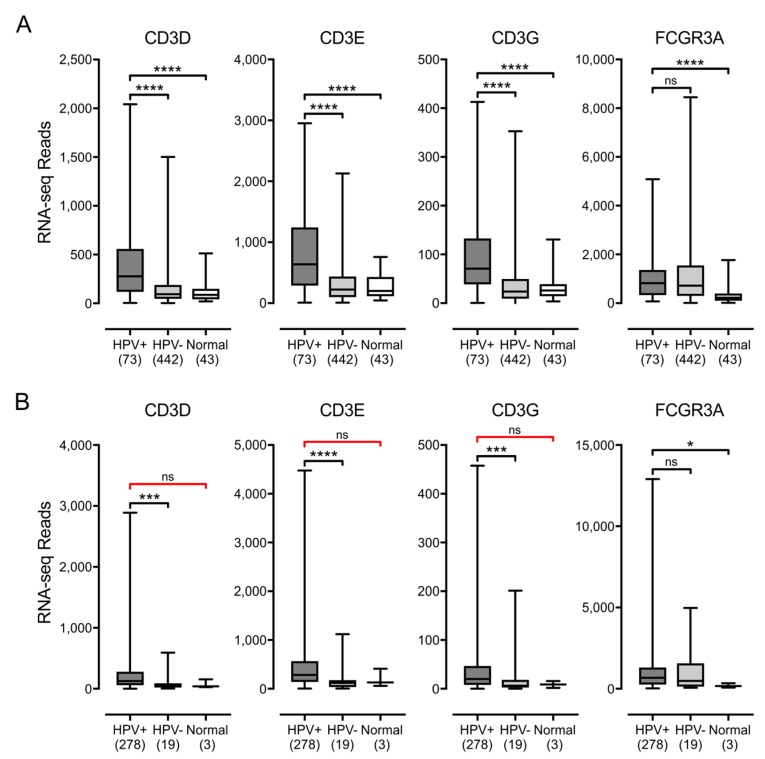
Detection of tumor infiltrating T-cells and NK-cells in head & neck and cervical carcinomas stratified by HPV status. Normalized RNA-seq data for genes indicative of tumor-infiltrating T cells (CD3D, CD3E and CD3G) or NK cells (FCGR3A) was extracted from the TCGA database for the HNSC (**A**) and CESC (**B**) cohorts for HPV+, HPV−, and normal control tissues. Numbers in brackets refer to the number of samples included in each analysis. * *p* ≤ 0.05; ** *p* ≤ 0.01; *** *p* ≤ 0.001; **** *p* ≤ 0.0001; ns—not significant; red brackets indicate a comparison that did not achieve significance with a power value <0.8.

We first assessed the relative proportion of T- and NK-cells in these samples, which are the primary producers of IFN-γ. Similarly to what has been done by others [54], we analyzed the mRNA expression levels of the genes encoding CD3 (CD3D, CD3E and CD3G) and CD16a (FCGR3A), as surrogate markers for T- and NK-cells, respectively (Figure 5). HPV+ samples from both the HNSC and CESC cohorts showed significantly increased expression of all three genes encoding subunits of CD3 versus HPV− samples. For the HNSC cohort, CD3 expression in HPV+ samples were also consistently higher than normal control tissues. No significant differences in CD3 levels were observed between HPV+ samples and normal control tissues in the CESC cohort, but this comparison was not powered sufficiently to confirm this result. For the NK-cell marker CD16a, expression of FCGR3A was readily detected and significantly higher in HPV+ cells versus normal control tissue from both the HNSC and CESC cohorts. No difference in FCGR3A levels were apparent between HPV+ and HPV− samples from either cohort, which indicated that infiltration by NK-cells is similar in both HPV+ and HPV− samples. Taken together, these results suggest that there is significant infiltration of both cervical and head & neck tumors with T-cells and likely NK-cells, which could be stimulating the tumor cells to upregulate MHC-I. Notably, the absolute normalized RNA-seq read levels for genes derived from these infiltrating lymphocytes were much lower than any of the other genes we analyzed, with the exception of the weakly expressed HLA-G. This was as expected, as the lymphocytes are expected to represent only a fraction of the tumor mass [55].

**Figure 6:**
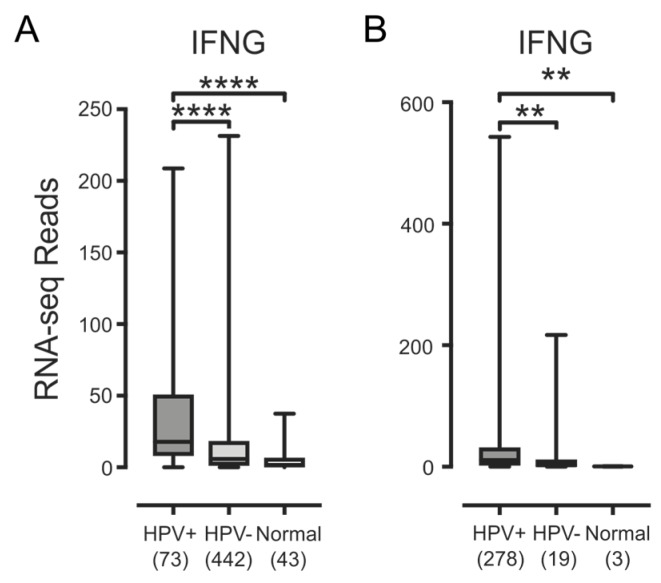
Detection of IFN-γ mRNA in head & neck and cervical carcinomas stratified by HPV status. Normalized RNA-seq data for the IFN-γ gene produced by tumor-infiltrating lymphocytes was extracted from the TCGA database for the HNSC (**A**) and CESC (**B**) cohorts for HPV+, HPV−, and normal control tissues. Numbers in brackets refer to the number of samples included in each analysis. ** *p* ≤ 0.01; **** *p* ≤ 0.0001; ns—not significant.

We next looked for IFN-γ mRNA to determine whether these lymphocytes were producing this proinflammatory cytokine (Figure 6). Although the expression of IFN-γ mRNA in these samples was low, it was detectable, and significantly higher in HPV+ samples versus HPV− or normal control tissues in both the HNSC or CESC cohorts (Figure 6). Taken together, these results indicate that HPV+ carcinomas have a significant level of infiltrating lymphocytes that appear to be producing some IFN-γ. Thus, the upregulated expression of the MHC-I loading and presentation genes observed in these carcinomas may be a consequence of exposure to IFN-γ.

### 3.4. Genes Encoding Components of the Antigen Presentation Apparatus Are frequently Mutated in HPV+ Human Tumors

Precancerous cells undergo strong selection for properties that are advantageous for growth and survival, leading to clonal evolution and cancer formation. As such, tumor cells frequently acquire somatic mutations that activate oncogenes or inactivate growth inhibitory genes. In HPV-dependent cancers, the expression of virally-derived oncogenes should eliminate the need for somatic mutations in the key cellular regulatory pathways they target. Thus, if high-risk HPV E7 repressed MHC-I expression and function, the frequency of mutations in MHC-I that would allow tumor escape from the immune system should be reduced in HPV+ versus HPV− carcinomas.

**Figure 7:**
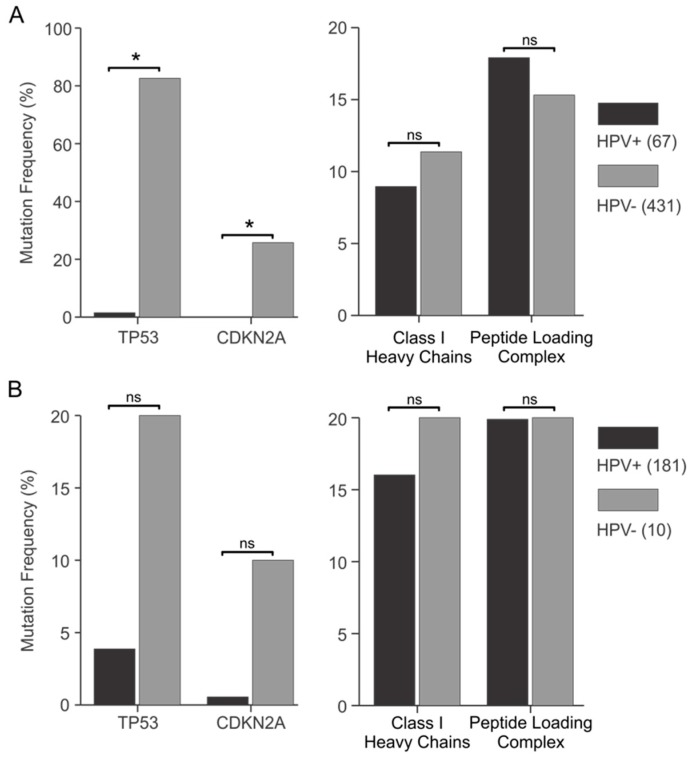
Comparison of the somatic mutation frequency in genes involved in MHC-I dependent antigen presentation in head & neck and cervical carcinomas stratified by HPV status. The presence or absence of somatic mutations in selected genes of the tumor samples in the TCGA HNSC and CESC cohorts was obtained from the Broad Genome Data Analysis Center’s Firehose server. The frequency of mutations in individual genes or groups of genes in HPV+ versus HPV− samples was calculated to determine whether they were influenced by HPV status. Analysis of TP53 and CDKN2A (left panels) revealed a significantly reduced frequency of somatic mutations in HPV+ versus HPV− samples in both the TCGA HNSC (**A**) and CESC (**B**) cohorts. No significant decrease was observed for somatic mutation frequency in the MHC-I heavy chain genes, or a collective grouping of all genes involved in MHC-I dependent antigen presentation between HPV+ and HPV− samples in either the TCGA HNSC (**A**) or CESC (**B**) cohorts. Numbers in brackets refer to the number of samples included in each analysis. * *p* ≤ 0.05; ns—not significant.

To validate this concept, we first analyzed the frequency of mutations in TP53 and CDKN2A (Figure 7A,B, left panels). TP53 encodes the p53 tumor suppressor protein, which is targeted by the E6 oncoproteins of high-risk HPVs. CDKN2A encodes the p16 tumor suppressor, a key regulator of the Rb pathway targeted by the E7 oncoprotein of high-risk HPVs [20,56]. There were dramatic differences in the frequency of somatic mutations in TP53 and CDKN2A in HPV+ versus HPV− samples from both the TCGA HNSC and CESC cohorts, although the frequencies in the CESC cohort were not statistically different due to a smaller sample size. The mutation load for TP53 was much higher in HPV− samples, with a 55-fold (HNSC) and 5.2-fold (CESC) increase compared with HPV+. For CDKN2A, a fold increase could not be calculated for the HNSC cohort, as no mutations were present in the HPV+ samples. However, 26% of HPV− HNSC samples contained CDKN2A mutations. In the CESC cohort, there was an 18-fold increase in CDKN2A mutations in HPV− compared with HPV+, respectively. These observations were expected, as high-risk HPV E6 targets p53 for degradation, while high-risk HPV E7 targets Rb. Thus, these viral oncoproteins functionally inactivate these critical anti-cancer pathways without the need for the somatic mutations acquired by HPV− carcinomas during tumor evolution.

We next compared the frequency of somatic mutations in all of the genes encoding MHC-I and the antigen-loading complex in HPV+ samples versus HPV− samples in the TCGA HNSC and CESC cohorts (Figure 7A,B, right panels). As noted by others [46,47], mutations in the genes encoding MHC-I heavy chains were the most frequent. However, mutations in B2M, TAP1/2, or other components of the MHC-I loading complex were also present at lower frequencies. To simplify this analysis, we compared the mutation frequency in both classical and non-classical MHC-I genes as a group, as well as all of the components of the MHC-I and associated loading complex collectively. This second comparison should be valid, as the inactivation of any single component of this pathway could be sufficient to impair antigen presentation [5]. Indeed, one comprehensive study in a small cohort of cervical carcinomas found that 15 out of 16 tumors exhibited an impaired expression of at least one component of the MHC-I antigen presentation apparatus [39]. Our inspection of the TCGA data reveals that both HPV+ and HPV− carcinomas in the HNSC or CESC cohorts acquire relatively frequent somatic mutations in the components of the MHC-I antigen presentation pathway, presumably as a means of immune escape. There was no difference in the frequency of somatic mutations in genes encoding MHC-I or the antigen-loading apparatus between HPV+ and HPV− samples in either cohort (Figure 7A,B, right panels). Thus, the presence of HPV oncogenes has no impact on the frequency of somatic mutations in genes related to MHC-I antigen presentation. This is consistent with the conclusion that HPV oncogenes do not effectively reduce antigen presentation by MHC-I, which would have eliminated the need for HPV+ carcinomas to acquire mutations in this pathway.

## 4. Discussion

The stable surface expression of MHC-I requires loading of the heavy chain/light chain dimer with an antigenic peptide in the endoplasmic reticulum. The loading complex consists of multiple essential factors, including the peptide transporter TAP, the bridging factor tapasin, and the chaperones: calreticulin, calnexin, and ERp57. Reduced expression or mutation of any of these components can compromise MHC-I dependent antigen presentation, and allow the infected or cancerous cell to escape T-cell recognition and clearance [5].

HPV infection and subsequent expression of the viral oncoproteins leads to a large-scale reprogramming of gene expression. Multiple studies have reported that the high-risk HPV E7 oncoprotein contributes to the evasion of the adaptive immune response; this process occurs through transcriptional downregulation of the MHC-I heavy chain genes or other components necessary for antigen loading and presentation [24,25,26]. This mechanism has been proposed to support the establishment of persistent HPV infection and contribute to the ability of HPV+ cancers to evade anti-tumor CTL responses. Additional downregulation of MHC-I is mediated by the high-risk HPV E5 oncoprotein. This occurs via a non-transcriptional mechanism in which the classical heavy chains are trapped in the Golgi apparatus and not transported to the cell surface [22].

While cell culture models have allowed detailed studies on the mechanism of E7-mediated transcriptional downregulation of components in the MHC-I antigen presentation apparatus, far less is known about how similar these effects are to actual HPV+ tumors. In this study, our goal was to determine whether the transcriptional reduction in MHC-I components mediated by E7 is recapitulated in primary HPV+ tumors from different anatomical locations.

Using data from over 800 primary human tumors, we provide evidence that HPV+ tumors from both the head & neck and cervical sites display high mRNA levels for MHC-I components, including heavy and light chains, as well as all factors required for MHC-I loading. Indeed, HPV+ head & neck carcinomas exhibit significant increases, rather than decreases, in mRNA levels for many MHC-I components compared with normal control tissue, and this trend continues in the cervical carcinoma cohort (Figure 1, Figure 2, Figure 3 and Figure 4). These unexpected results contradict the existing paradigm of virally-mediated immune evasion, and suggest that in the context of an in vivo tumor setting, the presence of HPV may actually result in enhanced expression of mRNA encoding MHC-I dependent antigen presentation components.

As our results specifically addressed the question of whether HPV oncoproteins repress expression of the MHC-I genes at the transcriptional level, they do not eliminate the possibility that other post-transcriptional mechanisms, such as those mediated by high-risk HPV E5, can reduce antigen presentation. However, a comprehensive analysis of MHC-I protein expression by immunohistochemical detection indicated that only a subset of about 40% of HPV+ cervical carcinomas have reduced expression of one or more components at the protein level [43]. As only a minority of carcinomas have reduced antigen presentation apparatus, it appears that the HPV-mediated reduction of MHC-I is not absolutely necessary for tumorigenesis.

Our results also do not rule out the possibility that E7 could mediate a reduction in MHC-I transcription in non-cancerous or pre-cancerous lesions, as all samples in this study are carcinomas. However, a purpose for the reacquisition of expression of MHC-I during the progression of a non-neoplastic lesion to a carcinoma is difficult to reconcile with current models of tumorigenesis.

Further evidence supporting our conclusion that HPV oncoproteins are unable to functionally antagonize MHC-I dependent antigen presentation in human carcinomas is provided by our comparison of somatic mutation frequencies between HPV+ and HPV− samples (Figure 7). The anticipated sharp reduction in the occurrence of mutations in genes encoding MHC-I or antigen-loading complex components was not observed in HPV+ samples. This is in stark contrast to what was observed for TP53, which encodes p53, a known target of the high-risk HPV E6 oncoproteins [17] or CDKN2A, which encodes the p16 component of the Rb pathway targeted by high-risk HPV E7 oncoproteins [20].

Furthermore, HPV utilizes multiple other strategies to evade the adaptive immune response, which suggests that a simple reduction in MHC-I transcription is unlikely to be sufficient to escape surveillance by the immune system. These include the ability of E5 to block MHC-I transport to the cell surface [22], the preferential utilization of low-abundance codons to ensure minimal translation of viral antigens [57], and the induction of the ERAP1 protease to partially degrade and attenuate the presentation of viral antigenic epitopes [58].

A simple mechanism to explain the lack of MHC-I repression would be a failure of these tumors to express the HPV E7 oncogene, which has been reported to be necessary and sufficient for the downregulation of transcription from at least some of these genes [24,25,26,27,28]. This is clearly not the case, as all HPV+ samples were reported to express detectable levels of E6 and E7 mRNAs [46,47]. In addition, a lack of E7 would not be expected to lead to the observed increase in MHC-I expression, but rather cause equivalent expression to that of HPV− cervical carcinoma samples.

An alternative method of activating expression of MHC-I and components of the antigen-loading complex could be via the activation of the JAK/STAT/IRF-1 signal transduction pathway by exposure to IFN-γ. However, multiple studies done in tissue culture models have shown that HPV E7 abrogates the IRF-1 response, thus inhibiting the IFN-γ induction of MHC-I expression, antigen presentation, and CTL-induced cell death [30,31,32,33,34]. Our analysis reveals low but detectable levels of mRNA for IFN-γ in HPV+ samples, which are higher than HPV− samples or normal control tissue in both TCGA cohorts (Figure 6). In addition, we searched for the presence of mRNAs that should be unique to infiltrating lymphocytes that can express IFN-γ (Figure 5). Using these surrogate markers, we found evidence for significant infiltration by T-cells and NK-cells in both HPV+ and HPV− carcinomas. Indeed, the levels of infiltrating lymphocytes were significantly higher in HPV+ vs. HPV− samples for both TCGA cohorts, which could have resulted from detection of foreign virally-derived antigens. Taken together, the high absolute levels of mRNAs encoding MHC-I components expressed in HPV+ head & neck and cervical carcinomas provide good evidence that high-risk HPV E7 is unable to efficiently block the IFN-γ induction of transcription of these genes in actual human tumors. Although this contrasts with the results of the studies listed above, our work agrees with other studies showing that various cervical cancer cell lines upregulate MHC-I in response to IFN-γ [59,60,61], and the treatment of HPV+ pre-cancerous cervical lesions in patients increased expression of at least HLA-B in a majority of cases [62]. It should be noted that the levels of MHC-I heavy chain mRNAs are statistically lower in HPV+ vs HPV− samples in the HNSC cohort (Figure 1), despite the increased levels of IFN-γ (Figure 7). This may indicate that HPV is able to modestly impact IFN-γ activation in these tissues, but this was not observed in the CESC cohort.

Our analysis of over 350 HPV+ head & neck or cervical carcinomas in comparison with over 450 HPV− cancers demonstrates subtle differences in the effect of HPV on MHC-I transcription between the two anatomical sites. Specifically, HPV does not repress the transcription of MHC-I heavy chain genes in cervical carcinomas, which exhibit high levels with respect to both HPV− carcinomas and normal control tissue. In contrast, a statistically significant but modest reduction in MHC-I heavy chain mRNA levels was observed in HPV+ vs HPV− head & neck cancers. However, transcription of these genes was nevertheless substantially elevated with respect to normal control tissues. Interestingly, our observations with the MHC-I heavy chain genes extend to many of the other genes involved in MHC-I loading. These results are in sharp contrast to much of the existing literature derived from tissue culture-based experiments, possibly because of the complex interplay between the MHC-I antigen presentation apparatus and cytokines produced by infiltrating lymphocytes present in actual tumors. This differs markedly from our previous study on the effects of HPV on numerous regulators of H3K27 methylation and the expression of multiple downstream targets of these regulators, in which we showed that tissue culture models faithfully recapitulate what is observed in human tumors [63].

Importantly, MHC-I dependent antigen presentation is critical for CD8+ T-cell responses, which are essential for the control and clearance of virally infected or cancerous cells. This is particularly important as recent advances in cancer immunotherapy using immune checkpoint blockades rely on reactivating antigen-dependent clearance of tumor cells [64]. Although we detect high levels of MHC-I mRNA in HPV+ tumors, this may not necessarily translate into high levels of expression of these proteins. In addition, a functional defect in any component of the MHC-I antigen presentation apparatus could compromise the detection and clearance of HPV+ carcinomas by the adaptive immune system [5]. Thus, the utility of immune checkpoint blockade therapy in HPV-dependent cancers remains to be determined. Nevertheless, it is intriguing to speculate that the expression of non-self derived viral antigens, combined with intact MHC-I presentation and increased levels of infiltrating T-cells, may contribute to the well-established observation that patient outcomes using conventional therapies are markedly better for HPV+ versus HPV− carcinomas in both the head & neck and cervix [49,65].
